# The Case for Advanced Recycling as a Path to Sustainable Food Packaging for Specialized Nutrition Products

**DOI:** 10.3390/foods14213586

**Published:** 2025-10-22

**Authors:** Melvin A. Pascall, Jameel Ahmed, Mary Beth Arensberg, Erica Ledbetter, Lauren Cheetham

**Affiliations:** 1 Department of Food Science and Technology, The Ohio State University, Columbus, OH 43210, USA; 2 Abbott Nutrition Division of Abbott, Columbus, OH 43219, USA

**Keywords:** food packaging, advanced recycling, sustainability, regulation, specialized nutrition products

## Abstract

Food packaging helps in labeling, transport, preservation, and the safety of food. Safety is especially critical in processing foods for vulnerable populations like infants/children, individuals with medical conditions, and older adults. These groups frequently rely on specialized nutrition products, including foods regulated in the United States (US) as infant formulas, medical foods, and foods for special dietary use (FSDU). As US states set post-consumer recycled (PCR) content mandates for product packaging, newer technologies like advanced recycling are essential to meet developing demands for recycled and sustainable packaging materials for specialized nutrition products. Also, advanced recycling must be fully supported in sustainability policies to ensure an adequate and safe recycled packaging supply. However, these urgent needs may not be well-recognized or understood. A literature search to identify scientific publications produced during the last 25 years found few papers specific to the packaging of specialized nutrition products and advanced recycling. Understanding emerging trends in safe food packaging materials, recycling, and sustainability policies is essential for maintaining access to specialized nutrition products in the US. This Perspective makes the case for advanced recycling as a path to safe, more sustainable food packaging for US specialized nutrition products and describes opportunities for strengthening the advanced recycling policy framework.

## 1. Introduction

Packaging is necessary for effective product storage, handling, and transport. For food, packaging is also important for product labeling, preservation, limiting food loss, as well as for safety. Maintaining safety is especially critical for the vulnerable populations who frequently rely on specialized nutrition products, including infant formulas, medical foods, and foods for special dietary use (FSDU). Emerging trends in sustainability policies and packaging materials in the United States (US) are converging with potential implications for the packaging of specialized nutrition products. Specifically, newer technologies like advanced recycling are essential to meet the developing demand for recycled packaging for specialized nutrition products. As a result, advanced recycling must be fully supported in sustainability policies to ensure an adequate supply of safe recycled packaging.

However, these urgent needs may not be well-recognized or understood, and the development of sustainable materials for food packaging may not be readily considered in the context of current sustainability policies. The goal of this Perspective paper is to explain the needs and share real-world evidence from the product manufacturing and policy framers’ points of view. The Perspective makes the case for advanced recycling as a path to develop more sustainable food packaging for US specialized nutrition products and describes opportunities for strengthening the advanced recycling policy framework.

As background, plastic is a versatile material commonly used for the packaging of food, including specialized nutrition products. Plastics regularly utilized in food packaging include single-layered polymers such as polyethylene terephthalate (PET), polypropylene (PP), and high-density polyethylene (HDPE) and low-density polyethylene (LDPE). Plastic food packaging can also be multilayered and hetero polymeric, with layers of different types of plastics, which can make it more difficult to recycle. Different from many other food products, multilayered plastic is a type of packaging material frequently used to maintain the safety of specialized nutrition products.

Waste recycling is fundamental to helping solve environmental issues and ensuring economic and ecological sustainability. As society grapples with the challenge of reducing the accumulation of discarded plastics, there is an increasing focus on the emerging trend of developing more circular pathways for used materials like packaging waste. Indeed, in the US, the Environmental Protection Agency (EPA) has recognized the recovery of discarded packaging as an important aspect of sustainable materials management [1].

Plastic food packaging waste can be recovered by recycling, downcycling, and/or processes that yield elements that could be converted to new plastic, fuel, or other sources of energy. Over half of the people in the US live in communities with curbside recycling programs. In over 80% of the US cities where this occurs, a single-stream collection system is used [2]. Plastic packaging and other materials collected in a single-stream system are not sorted until reaching a materials recovery facility (MRF). At these locations, the plastic materials are sorted and then sent to other facilities where they are processed via mechanical or advanced recycling methods.

Mechanical recycling is a physical process that shreds and extrudes used plastic into a post-consumer recycled (PCR) material that may be used in place of or mixed with virgin plastic. Advanced recycling is a developing industry that takes a different approach. Specifically, several distinct processes can be applied to break the chemical bonds of used polymeric plastic into basic chemical elements such as monomers, dimers, and trimers. These elements then become part of the pipeline components for making new virgin plastic and can be used to meet PCR requirements when certified as a mass balance allocation material [3]. Mechanical and advanced recycling are complementary processes. Both are necessary to create circularity for food packaging plastics, particularly when mechanical recycling cannot recover plastic materials efficiently and/or render them safe for food-contact use. Critically, applying the principles of a circular economy and promoting sustainability policies and actions requires finding solutions that can be customized to specific food packaging needs. This is why advanced recycling is an important pathway for sustainable food packaging of specialized nutrition products.

With greater plastic usage, waste disposal has continued to rise globally. In the US, plastic waste disposal rates increased nearly 20% from 2010 to 2018 [4], and now it represents the third largest category of waste disposal by weight (12.2%), behind paper/paperboard (23.1%) and food (21.6%) [5]. However, despite the urgent need to address packaging and other plastic waste globally, only 14% of plastic packaging is captured for recycling. An additional 14% is incinerated for energy recovery, 40% ends up in landfills, and 32% escapes the waste processing system [6]. The US has even lower recycling rates. In 2018, most US plastic went to landfills (76%), while smaller portions were incinerated (16%), and 9% was recycled [7]. More recently, US plastic waste recycling has further trended lower to just 5%, because of changes in international waste import markets and COVID-19 pandemic disruptions [8]. The pandemic also led to a 20–54% sales increase in processed and pre-packaged foods [9], compounding the problem. To support a circular plastics economy, more plastic waste needs to be diverted away from landfilling or incineration and towards recycling. Plastic food packaging plays a primary role in this since it accounts for the majority (50%) of the consumer packaging market, compared to the much smaller segments of consumer products (16.3%), healthcare products (14.6%), industrial products (9.8%), and other products (8.6%) [10].

Given the low US plastic recycling rates, it is not surprising that US state and federal governments are setting goals and implementing strategies to reduce the amount of plastic waste going into landfills. Many of these policy initiatives include establishing requirements for increased use of PCR in plastic packaging. Nonetheless, such mandates may not be realistic for specialized nutrition products if only mechanically recycled materials are recognized as PCR. The reason for this is the different types of manufacturing requirements. For example, for foods packaged in PET, mechanically recycled PCR may be an option because it is a low-diffusive polymer with relatively lower risk for contaminants [11]. However, mechanically recycled PCR is not optimal or safe for food contact with other types of plastic materials since they could be more prone to contain higher levels of contamination. But many of these plastics are required for product-specific, food-contact applications and sterilization processes used in manufacturing a number of specialized nutrition products. Thus, PCR mandates need to include provisions that can address safety and manufacturing requirements across the spectrum of today’s food product packaging. Further, there is a critical need to incorporate advanced recycling in governmental planning and in private investments in order to bring advanced recycling to scale.

In a previously published paper by several of the coauthors of this Perspective, the role and importance of functional food packaging for specialized nutrition products and sustainability implications for innovation and policy development were discussed [12]. This current Perspective builds on that work, and it focuses on a case for advanced recycling as a path to sustainable food packaging for specialized nutrition products. It also describes opportunities for strengthening the US advanced recycling policy framework. Specifically, it covers the following:

- The gap in published literature on food packaging of specialized nutrition products and advanced recycling;
- US regulations relevant to food packaging, PCR, and specialized nutrition products;
- Plastic materials commonly used in food packaging for specialized nutrition products and PCR concerns, including the potential for chemical migration;
- The basics of the US recycling process, mechanical and advanced recycling, and barriers limiting advanced recycling;
- Relevant and active areas of US legislation/regulation and policy actions needed to recognize and support advanced recycling to meet PCR mandates and the developing demand for recycled packaging for specialized nutrition products and other foods.

In addition, throughout this Perspective, specific implications for the packaging of specialized nutrition products and related sustainability policies are highlighted.

## 2. Literature Gap Related to Food Packaging of Specialized Nutrition Products and Advanced Recycling

Specialized nutrition products have distinctive and often more stringent regulations and food packaging material requirements when compared to other foods. At the same time, advanced recycling has qualities that make it a safe and viable pathway for packaging sustainability for such products and for meeting PCR requirements. A literature review outlining these unique opportunities is important to help inform and shape US sustainability policy for plastic packaging, so that its application to specialized nutrition products is not limited. Thus, in preparing this Perspective, an extensive background literature search was performed using PubMed to identify relevant publications produced over the past 25 years (2000–2025) on the subject of advanced recycling and food packaging for specialized nutrition products.

**Figure 1:**
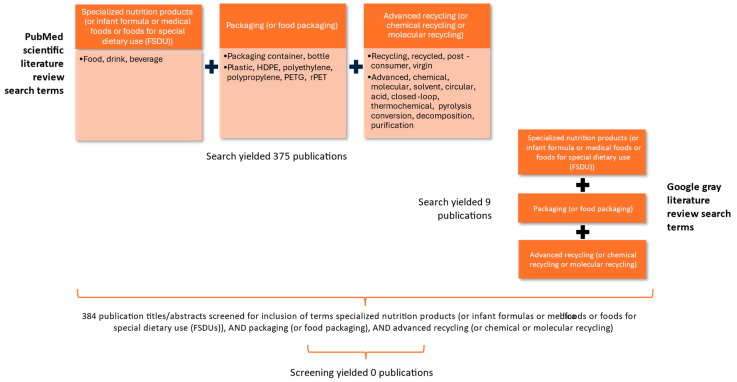
Search scheme of scientific and gray literature.

Figure 1 illustrates the strategy of our literature search and its focus on identifying scientific publications pertinent to the packaging of specialized nutrition products and advanced recycling. The primary search terms were specialized nutrition products (or infant formula or medical foods or foods for special dietary use (FSDU)), AND packaging (or food packaging), AND advanced recycling (or chemical recycling or molecular recycling). We also included other recognized and associated search terms. The PubMed search yielded 375 publications with no duplicates. To augment our findings, we used the same primary search terms to search the gray literature via Google and had a yield of 9 publications with no duplicates. Two co-authors then screened each of the 384 publications (titles and abstracts) to determine if they had inclusions of all three of the primary search terms.

Our findings showed that none of the publications contained all three of the primary search terms: specialized nutrition products (or infant formulas, medical foods, and/or foods for special dietary use (FSDUs)), packaging (or food packaging), and advanced recycling (or chemical or molecular recycling). Also, our previous publication [12], which focused on the food packaging of specialized nutrition products, did not meet the inclusion criteria since it did not address advanced recycling. These results should not be surprising, since specialized nutrition products, by design, meet the nutrition needs of only a small segment of the US food industry. For example, infants make up about 1% of the US population [13]. Further, advanced recycling is still an emerging recycling pathway in the US.

### Implications for Specialized Nutrition Products and Sustainability Policy

The null results of our review of scientific and gray literature point to a gap in publications that could help identify needs and opportunities for developing policies supportive of sustainable food packaging for specialty nutrition products. **Specific policy actions are needed to encourage research defining how to build the framework for advanced recycling and sustainable food packaging to serve all food products in the US**.

## 3. US Regulation of Food Packaging, PCR, and Specialized Nutrition Products

The US has federal regulatory frameworks specific to food packaging, to PCR use in food packaging, and to specialized nutrition products. Understanding these frameworks can be helpful in developing more sustainable food packaging. The regulatory structure for food packaging is based primarily on the US Food Additive Amendment of 1958, which requires the use of safe food packaging materials [14], and the US Food, Drug and Cosmetic Act that defines food packaging materials as food-contact substances [15]. Oversight for food packaging safety is the responsibility of the US Food and Drug Administration (FDA) and the United States Department of Agriculture (USDA), the federal agencies charged with regulating the safety and quality of most food sold in the US. Neither agency has responsibility for testing food-contact packaging to determine safety. This is the responsibility of packaging manufacturers, and it is described elsewhere in detail and in relation to specialized nutrition products [12]. At this present time, there are no active areas of policy change specific to the regulation of food-contact packaging.

When considering PCR plastic use in food packaging, it is essential to realize that PCR is a potential source for contamination. Potential contaminants carried in mechanically recycled plastic can include degradation products of polymers and additives, incidental contaminants from previous product use/misuse by consumers, cross-contamination from waste disposal, environmental contaminants, and plastic polymers entering the recycling stream that are not food-grade quality [16]. Moreover, while contamination from recycled plastic in food-contact packaging can become a critical public health and safety issue, the chemical safety of recycled materials in food packaging may be frequently ignored [17]. The FDA has identified its main safety concerns when PCR plastic materials come into contact with food as: “(1) contaminants from the PCR material may appear in the final food-contact product made from the recycled material, (2) PCR material not regulated for food-contact use may be incorporated into food-contact article, and (3) adjuvants in the PCR plastic may not comply with the regulations for food-contact use.” [18]. New food-contact packaging materials containing PCR, whether from mechanical or advanced recycling, must follow the same FDA approval process defined for all food-contact materials [19].

Notably, food-contact substances in packaging can potentially have an increased rate of impact on sensitive individuals versus the general population, depending on factors such as age, body size, metabolism, and/or medical conditions [20]. Such vulnerable groups can include those who use infant formulas, medical foods, and FSDU. Among the individuals in these groups are infants and children, older adults, and patients with acute conditions and chronic diseases such as cancer, diabetes, heart disease, gastrointestinal conditions, and malnutrition. The FDA has established definitions for infant formulas, medical foods, and FSDU (Table 1) and also has established regulations that include standards for food safety and quality, good manufacturing practices (GMPs), as well as specific requirements for nutrition, labeling, and claims that food manufacturers are required to follow. For example, among the FDA regulations and requirements for infant formulas are those related to product composition, labeling, GMPs, and pre-market notification of new or changed infant formulas (including review of infant formula packaging materials) [15].

### Implications for Specialized Nutrition Products and Sustainability Policy

There are specific US regulations for food packaging, PCR, and specialized nutrition products. The FDA has identified possible contaminants in PCR plastic used in food-contact applications as a safety concern. Material manufacturers have the responsibility for testing food-contact packaging to determine its safety and then seeking appropriate FDA approval for such materials. Some specialized nutrition products have additional regulations, such as a required review of food packaging materials for each individual infant formula product submission. This can make meeting PCR packaging mandates even more complicated, since all packaging changes would require resubmission of products for FDA review. **Improvements to sustainability policies should recognize the complexities of meeting safe packaging requirements for all food products, including those for advanced recycling and specialized nutrition products**.

## 4. Plastic Packaging Materials for Specialized Nutrition Products, PCR Concerns and Opportunities for Further Research and Development

Developing a more circular economy includes identifying sustainable food packaging solutions applicable to the spectrum of food products available in the marketplace. The expectations and requirements for the packaging of specialized nutrition products are very high compared to most other foods. To meet these challenges, packaging materials must fulfill multiple roles:

- Withstand manufacturing conditions such as sterilization/sanitization and high heat treatment/processing;
- Keep food products safe;
- Protect against potential chemical migration of toxic compounds—if present in the packaging material and they transfer into the food;
- Maintain food product nutrient levels (particularly important for vulnerable populations) [12].

Some of these roles, such as withstanding sterilization and high heat treatment, as well as adequately maintaining nutrient levels, are more rigorous for specialized nutrition products versus other foods, and this limits the types of plastic food packaging that can be effectively selected for these products.

### 4.1. Recycling Polyolefins

Polyolefins such as PP are the primary plastic used for packaging many specialized nutrition products because such materials meet the unique manufacturing requirements for these products. This can be quite demanding when retort processing is used [12]. However, when compared to other types of plastics, polyolefins can more readily degrade after use and recycling and can become a potential source for contaminant migration [23,24]. PP can be problematic to recycle mechanically due to compositional variability across waste streams, and this could cause inconsistent melt flow and processing behavior, unless the contamination is tightly controlled. For example, the tertiary carbon atom in the repeating monomeric structure of PP makes it highly susceptible to oxidative degradation during reprocessing, leading to chain scission and carbonyl group formation. This could serve to reduce molecular weight and alter the thermal properties of the polymer, thereby lowering its melting energy while increasing crystallization temperature and reducing the degree and/or size of the crystals. These changes, which can be further accelerated by contaminants, can compromise the mechanical performance of the polymer, and such recycled PP could show significant losses in tensile properties when compared to PP made from virgin resin [25].

As another contaminant-impacting example, during the mechanical recycling extrusion of HDPE, it is exposed to high-temperature processing under shear, which initiates chain scission and, in the presence of oxygen, promotes long-chain branching. This shift alters the polymer’s molecular architecture by increasing molecular weight, broadening its melting point distribution, and significantly affecting the material’s viscosity. These structural changes can result in HDPE with reduced mechanical performance when compared to its virgin resin [26].

Not surprisingly, most secondary post-consumer polyolefins rendered by mechanical recycling are not allowed for food-contact use [24], which limits their applicability as safe and approved PCR packaging materials. These constraints underscore the importance of recognizing advanced recycling—which does not face such challenges—as the primary path for incorporating PCR into food packaging materials for specialized nutrition products. Advanced recycling is not without constraints though, because it can be a complex manufacturing process. Polyolefins like PP and HDPE are chemically inert, and they are formed through the addition polymerization process. Reversing them to their monomeric states demands a substantial Gibbs free energy change (i.e., energy needed for spontaneous reactions to occur), and this results in the formation of complex mixtures of chemical products during the pyrolysis process. Advanced recycling via pyrolysis converts plastics into a mixture of oils and waxes, which then requires additional treatment steps such as hydrotreatment, to yield purified monomers suitable for new polymer production [27]. The process is, thus, energy-intensive, and it requires the proper handling of waste chemicals.

### 4.2. PCR Contamination

As discussed earlier in this Perspective, advanced recycling offers a feasible pathway to meet the stringent regulations and requirements when PCR is utilized in food-contact applications, particularly for specialized nutrition products. In contrast, the use of mechanically recycled PCR in food-contact applications poses multiple challenges, including the issue of contamination that may occur during the mechanical recycling process. Indeed, a workshop on Scientific Advances and Challenges in Safety Evaluation of Food Packaging Materials concluded, “The possibility that contaminants in plastic materials intended for recycling may remain in the recycled material and could migrate into food, is one of the major considerations for the safe use of recycled plastics, illustrating the need for the development of new recycling technologies” [28].

Unlike virgin polymers that originate from new raw materials, mechanically recycled PCR polymers are an output from previously used materials, and this prior usage/misuse can expose the polymers to different chemical contaminants. Moreover, the mechanical recycling process can add or concentrate specific compounds, including plastic additives or contaminants, in PCR polymers. Thus, mechanically recycled polyolefins are more prone to cause higher levels of chemical contaminant migration from plastics to packaged foods when compared with virgin and/or advanced recycled materials. In support of this, Tumu et al. [29] reported the presence of phthalates and polyfluoroalkyl substances (PFAS) in PCR polyolefins but not in the virgin counterparts. In their discussion, they suggested that the possible contamination source was the recycling process, potentially due to degradation products or from processing aids, and/or previously unknown applications of the original materials prior to processing.

Plastic often contains additives like plasticizers, stabilizers, antioxidants, flame retardants, colorants, and antimicrobials that are included to enhance or modify the plastic’s properties for specific applications. During recycling--particularly mechanical recycling--these additives are not easily removed, and, therefore, they can be present in the recycled plastic, becoming “legacy additives.” The concern with legacy additives in food-contact packaging is their potential to migrate into the food from the plastic packaging, posing health and safety risks [30].

Another challenge is contamination by way of non-intentionally added substances (NIAS). These are substances occurring from unintentional processes during the lifecycle of the plastic, and these are more likely to arise during recycling. NIAS can result from chemical reactions at various stages during the recycling process, when the degradation of additives can occur. For example, components such as bisphenol A (BPA) or bisphenol S (BPS) are not used in the manufacture of polyolefins but can be found in the environment and potentially can be in certain food-contact resins and coatings. In one study of bisphenols in recycled plastics, Nunez et al. [31] identified low concentrations of BPA and BPS in virgin plastic samples but higher concentrations in recycled plastic of the same type. The results also showed that a significant portion of the BPA in the recycled samples was NIAS [31]. Other types of NIAS include contamination from inks, adhesives, or residues from previous contents of the recycled plastic, and other reactions between products and impurities during the original production or recycling process. Consequently, the use of mechanically recycled PCR plastic in food-contact applications raises concerns, as it can be a direct pathway for NIAS migration into packaged food [16].

Mechanical recycling is a physical process that shreds and extrudes used plastic into PCR material. However, research shows the shredding stage can unintentionally generate microplastics, and the amount produced varies by polymer type and hardness. For example, polycarbonate (PC), a relatively harder and more brittle plastic, can generate up to 3.3 times more microplastics than HDPE during shredding, with a strong correlation found between material hardness and microplastic generation [32]. The advantage of advanced recycling is that, through depolymerization, plastics are chemically broken down into their fundamental monomeric building blocks. This eliminates the polymer structure that fragments into microplastics, thus reducing the risk of microplastic formation during the recycling process [33].

### 4.3. Opportunities for Further Research and Development

Along with incorporating PCR into food product packaging, another way to support a circular economy is to design the initial use package with features that encourage recycling and the product’s end-of-life disposition process [34]. This presents an opportunity for further research and development. Indeed, there continues to be a focus on identifying safe, environmentally friendly, and biodegradable packaging that meets manufacturing constraints and consumer expectations.

In the paper by Pascall et al. [12], it was explained that waste reduction strategies must be balanced against the needs of specialized food product packaging. These needs include nutrition integrity, quality, safety, product functionality, and the necessary regulatory requirements. Thus, while there are regulatory pathways for seeking food-contact use approval of new materials, additional scientific investigation is needed to determine commercial viability for individual products and then investments to bring these innovations to scale. Pascall et al. [12] identified multiple innovations and potential applicability/limitations for specialized nutrition products; examples from their work and other researchers are summarized in Table 2.

### 4.4. Implications for Specialized Nutrition Products and Sustainability Policy

Microplastic formation, NIAS reactions, polymer/additive degradation, and incidental and cross-contamination are among the challenges associated with mechanically recycled PCR. Such contamination can lead to human health and safety risks. For specialized nutrition products, advanced recycling is the safest and most viable option to limit contamination and meet PCR requirements. Innovations generated from emerging food packaging frontiers may be beneficial in the long term for helping specialized nutrition products meet such challenges if the innovations become commercially viable and align with specific product requirements. **In the short term, it is critical for advanced recycled materials to be recognized as a viable PCR option in packaging policy mandates.**

## 5. The US Recycling Process

Understanding the US recycling process helps provide context for the US policy landscape described in the next section of this Perspective. The International Organization for Standardization (ISO) defines the recycling process as a “physical or chemical process which converts collected and sorted used packaging together, in some instances with other material, into secondary (recycled) raw materials, products, or substances, excluding energy recovery and the use of the product as a fuel” [41]. Building on this, the US recycling system has three basic elements: collection, processing, and remanufacturing into new products. The MRF has responsibility for multiple activities, which can be categorized into two primary steps:

- Sorting at the MRF, where recyclables are processed by removing physical contaminants (like trash) and then separating them into paperboard, paper, metal, glass, and plastic materials by a variety of methods, depending on the capabilities of the individual MRF;
- Compacting and preparation for transportation, where the materials are compacted into bales, palletized, or otherwise placed into containers and sent to individual recycling facilities that process each material by type.

Plastic bales can vary in quality, density, and composition depending on plastic type. The goal of MRFs is that bales contain the most potentially recyclable materials and the least amount of non-recyclable residues. The Association of Plastic Recyclers (APR) provides bale specifications that establish quality standards, set a minimum bale density of 240 kg/cm^3^, and limit total contamination to 6% for thermoformed PET bottles, 5% for HDPE colored bottles, and 15% for PP bottles [42].

### 5.1. Mechanical Recycling

Amongst various recycling methods, the most common approach is mechanical recycling, and it is well-established in many countries. The US follows a mechanical recycling process that is similar to those in other countries, and it involves collecting, sorting, or taking sorted bales for further sorting to increase yields and reduce contamination. It also includes the washing, grinding, melting, and extruding of the recycled materials into resin pellets. The resin pellets can then be reused in manufacturing processes, such as product packaging. Mechanical recycling works well for homogenous plastic streams like clear PET water bottles or HDPE milk bottles, in which the recyclate is most often incorporated into non-food packaging applications.

Reclaimers primarily use household waste as their source for mechanical recycling. Household plastic packaging waste usually consists of a diverse and heterogenous mix of plastic [43]. Even with improvements in material sorting and segregating technologies, mechanically recycled post-consumer materials—especially polyolefins—may exhibit reduced/variable physical, mechanical, and processing properties (by up to 10% for each cycle) [24]. This limits its lifecycle because of the broad spectrum of molecular weights, polymeric structures, and additive components that can be present in these packaging materials. The process is referred to as “downcycling” since each time a given material is recycled, it loses some of its essential functional properties [24].

### 5.2. Advanced Recycling

Advanced recycling, also called chemical or molecular recycling, can help overcome such limitations and can help to ensure a high-quality plastic, while maintaining virgin plastic resin-like properties that are suitable for food-contact packaging. The term advanced recycling has not been consistently defined but typically describes several different classes of plastic reprocessing and recycling technologies. Advanced recycling is not a substitution for but rather a complementary process to mechanical recycling, since hard-to-recycle plastic streams, which otherwise would be incinerated or landfilled, can be recycled.

Advanced recycling breaks down plastic into its basic resin components, which can then be incorporated back into the production of plastic, replacing the fossil sources required to generate new virgin plastic resin. Several studies have indicated that if advanced recycling technologies were to be widely adopted, they could potentially increase the global plastic packaging recycling rate to 50% by 2040 [44,45].

The three common and commercially most predominant processing technologies for advanced recycling are conversion, depolymerization/decomposition, and purification/dissolution [7]. There are variations in the ways these technologies may be applied as well as the end products produced. Overall, advanced recycling is a more complicated and energy-intensive process than mechanical recycling, and the yields are lower as well. There is the potential implication that the plastic from advanced recycling will then come at a cost premium when compared to the plastic from mechanical recycling, but simple comparisons can be difficult to make if the environmental and economic benefits are not considered. The mechanical recycling process is straightforward, whereas advanced recycling requires the use of different and emerging technologies, some of which have been more researched than others [46]. One of the advantages of advanced recycling is the fact that it is capable of processing heterogeneous mixes of plastics and can convert them into high-quality polymers. Thus, advanced recycling is more capable of diverting plastic waste from landfills and incineration when compared to mechanical recycling [45].

### 5.3. Barriers to Advanced Recycling

Advanced recycling is still an emerging industry in the US, and it is not yet at a scale to meet the needs of food manufacturers. Barriers that limit its contribution in an impactful way to the circular plastics economy include its high cost, heterogenous industry standards, government policy, and consumer behavior. Currently, advanced recycling technologies are in different stages of commercial maturity, and these have an impact on throughput, capital, and operational costs. Large-scale investments in research and technology development, waste collection, partnerships, and key infrastructures are necessary to bring advanced recycling fully to the fore [45]. Further, methods standardization and certification, and traceability frameworks are important and require additional development by a cross-functional network of stakeholders. Paths for increased circularity for plastics in the US have been identified, and researchers have noted that while scaling up sorting infrastructure and new recycling facilities will not be simple, development and commercialization are underway to produce higher-quality PCR from mixed plastic waste [47].

### 5.4. Implications for Specialized Nutrition Products and Sustainability Policy

Single-stream recycling is the primary collection system in the US; recycled materials are not sorted until they arrive at MRFs, and there is no separate stream for specific materials, like plastic food packaging. Advanced recycling is more viable than mechanical recycling in reducing contamination in PCR plastic for food packaging, and advanced recycling is the primary and safer path for specialized nutrition products to meet PCR mandates. **There is a need for policies that encourage standardization of advanced recycling methods, the development of certification measures for facilities that meet certain minimum thresholds, and traceability frameworks**.

Stakeholder education for both policymakers and the public at large on how advanced recycling complements mechanical recycling in the PCR pathway is essential. Such education can help build a strong policy foundation that supports advanced recycling as part of sustainability-related legislation/regulation and provides a safe and effective path for food and specialized nutrition products to meet PCR packaging mandates. **As part of this foundation is the need to incentivize investments in advanced recycling technologies and related key infrastructures**.

## 6. US Policy Landscape and Advanced Recycling

The US landscape for environmental and sustainability policy to build a more circular economy for plastic packaging is continuing to evolve. Complimentary policy solutions are needed to emphasize reduction and safe reuse of plastic packaging materials as well as policies that support the circular economy and further the development of recycling technologies, such as advanced recycling of plastic materials. In the US, this policy framework is shaped by actions at both local and national levels. States are taking the lead, with legislation and regulation, which impact both specialized nutrition products and advanced recycling, as described in further detail below.

### 6.1. US State Legislation and Regulation

The primary method used by states within the US to emphasize the reduction and reuse of plastic food packaging is through the enactment of extended producer responsibility (EPR) and PCR legislation. The EPR legislation has included language that shifts the responsibility for packaging waste from the states and consumers to the product producers. The goal is for producers to be accountable for product-related waste throughout their products’ lifecycles. Further, EPR policies can support government, community, and producer recycling and materials management goals that together help build a more circular plastics economy and can also encourage development of recycling technologies like advanced recycling.

Seven US states have passed EPR legislation related to food packaging. These are the states of Maine, Oregon, California, Colorado, Minnesota, Washington, and Maryland [48,49,50,51,52,53,54]. The Colorado, Minnesota, and Washington EPR bills mandate their EPR programs to set minimum recycled content requirements for plastic packaging. Separate PCR bills have recently been signed into law as well, and they typically include targets that increase over time for the percentage of PCR plastic in specific materials. The states of California, Washington, Maine, New Jersey, and Connecticut have passed PCR bills specific to plastic beverage containers [55,56,57,58,59]. Most of the US EPR and PCR bills include exemptions for infant formulas, medical foods, and FSDU or specific types of FSDU (e.g., fortified oral nutrition supplements for those needing supplemental/sole source nutrition because of certain medical conditions). Including such exemptions in future EPR and PCR bills is essential to assuring the continued availability of specialized nutrition products for the vulnerable groups who need them.

EPR, PCR, and other environmental and packaging-related legislation/regulation are driving a growing demand for PCR plastics. However, without concomitant policy development for expanding and encouraging advanced recycling, there are few safe options for food-contact applications. Since 2017, 27 US states have passed bills designating advanced recycling as a type of manufacturing and exempt from municipal solid waste facility requirements [60], including bills more recently enacted in the states of Louisiana, Michigan, Indiana, Utah, and Wyoming [61,62,63,64,65]. This is an important first step. In contrast, three states, Maine, New Jersey, and New Mexico, have enacted regulations or statutes that identify pyrolysis of waste plastics as a form of solid waste disposal, imposing substantial regulatory burdens [66,67,68]. The sustainability policies of all US states, either as part of EPR, PCR, or advanced recycling legislation or regulations, should include considerations for the mass balance of the chemical content of a given plastic packaging material. Mass balance is a scientific, chain-of-custody accounting method that traces the flow of a material through a supply chain, allowing recycled packaging materials from advanced recycling to be mixed with virgin materials, while certifying that a specific portion of the material can be traced and attributed to specific end products. Third-party independent certification and strict documentation are critical to ensuring the integrity, transparency, and credibility of mass balance claims throughout the supply chain [69]. Recognition of mass balance certification is important for manufacturers to effectively utilize advanced recycling to meet PCR packaging requirements.

Table 3 summarizes the sustainability policies that impact food packaging in various US states. It also includes the potential limitations of legislation/regulation specific to EPR, PCR, advanced recycling facility classification, and mass balance methods. Notwithstanding these limitations, US states are encouraged to continue to invest in infrastructure that helps to increase recycling rates. In addition, as states continue to pass packaging and packaging waste legislation, the definition and inclusion of advanced recycling as a method to help achieve PCR requirements are imperative for specialized nutrition products. Nonetheless, to date, the development of advanced recycling has not been a focus of some US state bills.

### 6.2. US Federal Legislation and Regulations

In general, legislation and regulations at the US federal and state levels are not as forthcoming when compared with similar actions from European Union legislators. Although federal Congressional action on advanced recycling has been slow to take hold, it has recognized the overall need for recycling infrastructure development. Advanced recycling was specifically included in the Accelerating a Circular Economy for Plastics and Recycling Innovation Act introduced in 2024, which identified advanced recycling as a “critical component” of the US National Recycling Strategy [70]. This bill emphasizes the need to modernize the US recycling infrastructure, and it allows for new technologies to help address plastic waste. It also calls for 30% PCR in plastic packaging by 2030. Significantly, the bill recognizes the importance of an adequate and safe supply of PCR, including for the packaging needs for specialized nutrition products. It also allows for adjustments to the minimum percentage of PCR based on the “supply of on-specification recycled feedstocks available for mechanical or advanced recycling to be used in plastics packaging that contains…medical food, or infant formula … or any other product packages with health and safety related recycled plastics restrictions” [70].

The Accelerating a Circular Economy for Plastics and Recycling Innovation Act has not yet been reintroduced in the current Congress. Several other recycling infrastructure bills have been introduced, although they do not specifically mention advanced recycling. These new bills include the following:

- Cultivating Investment in Recycling and Circular Local Economies (CIRCLE) Act of 2025 that incentivizes domestic recycling investment by proposing a 30% tax credit over 10 years for qualified investments in new or upgraded recycling infrastructure [71];
- Strategies to Eliminate Waste and Accelerate Recycling Development (STEWARD) Act of 2025, which establishes a federal grant program supporting state and local recycling programs, enhances public education, and promotes market development for recycled materials [72];
- Recycling Infrastructure and Accessibility Act (RIAA) of 2025 that expands recycling services in underserved and rural communities, through funding of infrastructure improvements to increase curbside/drop-off access [73].

Federal agencies have included several comments over the past two administrations specific to advanced or chemical recycling and to the packaging needs of specialized nutrition products. As an example, the final 2021 EPA National Recycling Strategy, in discussing stakeholder comments received on a draft of the Strategy stated “All options, including chemical recycling, should be discussed when considering methods for sustainably managing materials. Therefore, chemical recycling is part of the scope of this strategy, and further discussion is welcome” [74]. Also in 2021, the Government Accountability Office (GAO) commented advanced recycling, like chemical recycling technologies, “have the potential to improve plastic recycling” [7]. Specialized foods were mentioned in the 2024 EPA National Strategy to Prevent Plastic Pollution when it acknowledged that concerns had been expressed about decreasing access to single-use plastic packaging and that this could potentially result in unintended consequences, including the “ability of alternatives to meet the requirements of specialized food and medical packaging” [75]. However, in general, the need exists for more federal regulations with the intent to positively impact the advanced recycling industry by influencing opportunities to build new sources of PCR materials. Consistency in regulatory approach is one area to address, as described in the two examples discussed below.

The US EPA has oversight of advanced recyclers’ environmental impact under the Toxic Substances Control Act (TSCA) [76] and the Clean Air Act (CAA) [77]. As part of TSCA, advanced recyclers are required to submit premanufacture notifications (PMNs), and the EPA then performs a risk review. The EPA risk reviews have typically focused on workers’ and transportation health and safety needs. In 2023, the EPA proposed Significant New Use Rules (SNURs) expanding requirements for advanced recyclers seeking to produce 18 chemicals already approved under previous PMNs. These 2023 SNURs were later withdrawn in 2025, signaling the evolving nature of EPA regulations of advanced recycling, which can make it more difficult for advanced recyclers to appropriately plan for the regulations they will be required to follow.

EPA positions have also shifted over time on whether advanced recycling pyrolysis is subject to EPA New Source Performance Standards (NSPS) under the CAA. Two decades ago, the EPA position was that advanced recycling pyrolysis of plastic waste was regulated under NSPS. In 2020, the EPA issued a proposed rule reversing this position and potentially decreasing regulatory burden. The EPA withdrew its 2020 proposed revisions in 2023, thus reverting to the original 2005 EPA interpretation that advanced recyclers must continue to meet NSPS under the CAA. No further action has been taken since that time. However, an urgent need exists for EPA regulations to be consistent in approach so advanced recyclers can design and build their processes accordingly to better help meet the needs of food manufacturers seeking reliable sources of food-contact safe PCR materials. Table 4 summarizes the potential limitations for advanced recyclers with these federal sustainability policy shifts.

### 6.3. Implications for Specialized Nutrition Products and Sustainability Policy

The US policy landscape for advanced recycling is in an evolutionary phase. However, the urgent need exists for less variability in US state-by-state EPR and PCR legislation/regulations. **Including exemptions for products like specialized nutrition products is critical because the supply of food-contact safe PCR from advanced recycling is still in the developing phase**.

Advanced recycling development can be impacted by how states and the federal government classify advanced recyclers, and the pyrolysis process itself. **Consistency in policy approach at both the state and federal levels is needed to expand and encourage the development of newer technologies like advanced recycling**.

The federal Accelerating a Circular Economy for Plastics and Recycling Innovation Act introduced in 2024 [70] can serve as a model for future legislation and other US policy actions. It can also serve as a template to support further development of advanced recycling technologies and their food-contact applications, including those for specialized nutrition products. **Progress will require value chain collaboration and agreements between technology providers, resin producers, waste management companies, and food-producing companies**.

## 7. Conclusions

The US has specific federal regulations for food-contact applications, PCR, and specialized nutrition products that make meeting PCR requirements for food packaging of specialized nutrition products challenging. Advanced recycling can provide a sustainable food packaging opportunity for these products to continue to support vulnerable populations. This Perspective sought to help address the gap in published papers on the subject of advanced recycling and specialized nutrition products by describing the product manufacturing process, the policy environment, and recent trends related to these areas. Limitations of this Perspective include that it is US-focused and is not a data-driven research study. It acknowledges that further investigation on the subject matter is needed. Nonetheless, in making the case for advanced recycling to help develop more sustainable food packaging for specialized nutrition products, the Perspective also identified opportunities to strengthen the US advanced recycling policy framework. These policy opportunities include the following:

- Encouraging research to define opportunities for building the US policy framework for advanced recycling;
- Recognizing advanced recycling as a path to addressing the complexities of food-contact safe packaging;
- Including advanced recycling as a method to achieve food-contact safe PCR requirements;
- Encouraging methods standardization, development of certification, and traceability frameworks for advanced recycling;
- Incentivizing investments in advanced recycling technologies and key infrastructures to build scale;
- Including exemptions when the supply of food-contact safe PCR from advanced recycling is limited;
- Taking a consistent approach to legislation and regulation of advanced recycling to encourage its development;
- Building cross-functional advanced recycling stakeholder networks and value chain collaborations.

Such information, as provided in this Perspective, can help inform and shape research agendas and US policy for plastic packaging, so that it better supports the development of sustainable materials in food packaging and their application to specialized nutrition products. Ultimately, increasing understanding of emerging trends in safe food packaging materials and recycling, and the need for policy actions, are essential in helping to maintain access to specialized nutrition products in the US.

## Figures and Tables

**Table 1:** US federal regulatory categories and statute or regulation numbers and definitions for specialized food products.

Federal Regulatory Category	Federal Statute or Regulation Number and Definition
Infant Formulas	21 USC 321 (z): A food which purports to be or is represented for special dietary use solely as a food for infants by reason of its simulation of human milk or its suitability as a complete or partial substitute for human milk [15]
Medical Foods	21 USC 360ee (b) (3): A food which is formulated to be consumed or administered enterally under the supervision of a physician and which is intended for the specific dietary management of a disease or condition for which distinctive nutritional requirements, based on recognized scientific principles, are established by medical evaluation [21]
Foods for Special Dietary Use (FSDU)	21 CFR 105.3: The term special dietary uses, as applied to food for man, means particular (as distinguished from general) uses of food, as follows: (i) Uses for supplying particular dietary needs which exist by reason of a physical, physiological, pathological, or other condition, including but not limited to the conditions of diseases, convalescence, pregnancy, lactation, allergic hypersensitivity to food, underweight, and overweight; (ii) Uses for supplying particular dietary needs which exist by reason of age, including but not limited to the ages of infancy and childhood; (iii) Uses for supplementing or fortifying the ordinary or usual diet with any vitamin, mineral, or other dietary property. Any such particular use of a food is a special dietary use, regardless of whether such food also purports to be or is represented for general use [22]

**Table 2:** Recycling and waste reduction strategies and challenges and limitations for specialized nutrition products.

Recycling and Waste Reduction Strategies	Challenges and Limitations for Specialized Nutrition Products *
Reducing packaging materials	Manufacturing process requirements can limit some source reduction opportunities; for example, the heat of retort processing requires the use of containers heavier in weight when compared to the same container used for cooler processing conditions [12]
Recycling packaging materials	Post-consumer recycled (PCR) polyolefins are at higher risk for degradation and contamination uptake; most are not currently approved for safe food-contact use [12]
Incorporating biobased, edible, and nanotechnology packaging	Biobased packaging has limited applications due to lower barrier properties, weaker physical protection/mechanical strength [35], and heat instability [36] when compared to traditional petroleum-based plastics Certain biobased packaging materials have potential applications when strength/stability requirements are lower, such as in lids and scoops used with powdered infant formula products [37] Edible films, compared to petroleum-based polymers, have poor mechanical properties, weak resistance against water/gases, and insufficient physical properties, which can limit their applicability [38] Application of nanotechnology has limited published research on its toxicological effects and its influence on the biodegradability of packaging materials [38]
Active packaging (extends shelf life by incorporating active compounds to slow microbial growth and enhance product quality/safety)	More research is needed to determine the feasibility of use and the development of materials that can withstand manufacturing rigors [12]
Intelligent packaging (uses technology to provide product information, including information about quality and traceability, which can help reduce food waste)	For physical monitoring systems, further research and development are needed to bring prototypes to the commercial scale and to reduce the cost of material accessories (indicators, sensors, and data carriers) and facilitate changes to processing operations [39] Innovations in product label technology (e.g., quick response (QR) codes) and others such as artificial intelligence (AI) are already being used to provide greater product transparency and information on supply chain movement [40]

* Based on current commercialization and availability of materials/technologies.

**Table 3:** Summary and potential limitations of some US state sustainability policies that impact food packaging.

State Sustainability Policies	Description	Responsibilities of Food Manufacturers and Advanced Recyclers	Potential Limitations for Food Manufacturers and Advanced Recyclers
Extended producer responsibility (EPR) legislation	It shifts responsibility for funding the collection, recycling, and end-of-life of packaging from municipalities/taxpayers to producers Most EPR packaging programs require producers to join collective producer responsibility organizations (PROs), which develop/manage programs EPR bills generally require PRO fees to cover the management of used packaging PRO fees may also include covering costs for consumer outreach/education and expanded curbside service to increase recycling rates, infrastructure improvements, and end-market development of recycled materials	Food manufacturers pay fees to PROs, which fund program costs required by EPR legislation	EPR and PRO requirements and fee structures vary by state or are non-existent in some states Differing definitions and costs mandated for coverage create a patchwork of requirements that are a burden to food manufacturers Only some state EPR bills address infrastructure development, potentially limiting the ability to bring advanced recycling to scale
Post-consumer recycled (PCR) material legislation	Refers to materials recycled from used consumer packaging that has been collected and recycled Establishes minimum required PCR content in product packaging Laws typically include provisions that set mandates for future years with increased percentages over time for required PCR content in packaging materials	Food manufacturers typically must meet percent required PCR content in their packaging materials, or pay a fee, or discontinue selling product in the state	PCR requirements and timeframes vary by state or are non-existent in some states and may not consider advanced recycling as a pathway to meet PCR requirements Mechanically recycled PCR is generally not approved for food-contact use, is often contaminated with various materials that can be difficult to eliminate, and may be at higher risk for degradation; all of these are factors impacting food manufacturers’ ability to meet state PCR requirements
Advanced recycling facility classification legislation/regulation	Classify waste plastic pyrolysis as either a manufacturing or waste disposal process	Advanced recyclers classified as solid waste processing facilities face more regulations, some of which may not be applicable	Classification of advanced recycling facilities varies by state or is non-existent in some states Classification of advanced recyclers as solid waste processing facilities: Overlooks circular economy goals as advanced recyclers create new products for use rather than consolidating waste for disposal Undermines economic viability of advanced recycling technology, particularly when regulatory bodies apply outdated solid waste regulations Can mask environmental and resource impacts since advanced recyclers can use plastic waste energy during pyrolysis and limit use of natural resources to create new plastics as well as provide a recycle pathway for hard-to-recycle and mixed plastics versus discarding and landfilling
Mass balance accounting legislation/regulation	Scientific, chain-of-custody accounting methods for tracing a material’s flow through the supply chain Allows recycled packaging material from advanced recycling to be mixed with virgin materials and traced to finished packaging material May be included or excluded in state EPR, PCR, advanced recycling, and/or other sustainability legislation/regulation Some states allow use of mass balance to help meet PCR packaging requirements; other states do not	Food manufacturers must work with third-party certifiers to document and support mass balance claims	Policies for mass balance vary by state or are non-existent in some states Individual state classification of advanced recycling directly affects the use of mass balance principles States defining advanced recycling as waste disposal challenge food manufacturers’ use of mass balance to make PCR content claims for product packaging and meet PCR requirements

**Table 4:** Summary and potential limitations of US federal sustainability policy shifts impacting advanced recyclers.

Federal Sustainability Policies	Description	Responsibilities of Advanced Recyclers	Potential Limitations for Advanced Recyclers
Toxic Substances Control Act (TSCA) of 1976	US federal law granting the US Environmental Protection Agency (EPA) broad authority to regulate new and existing chemicals, for the protection of human health and the environment Pyrolysis outputs produced by advanced recycling are considered new chemicals because the chemical composition of waste plastics changes during the pyrolysis process	Advanced recyclers are required to submit Premanufacture Notices (PMNs) to the EPA for risk assessment of the chemicals they plan to produce, before production of those chemicals begins	Shifting policy approaches can lead to uncertainty in the EPA PMN risk assessment process, potentially limiting PMN approvals for new advanced recycling products, and this could be a challenge to more development occurring in the advanced recycling industry
Clean Air Act (CAA)	US federal law granting the EPA authority to establish and enforce air quality regulations	Advanced recyclers using pyrolysis must follow stringent new source performance standards (NSPS) under CAA, when their pyrolysis/combustion units are classified as other solid waste incinerators (OSWIs)	Shifting policy approaches in the classification of pyrolysis/combustion units as OSWIs and subsequent inclusion/exclusion in NSPS could be challenging to decision-making in the advanced recycling industry

## Data Availability

No new data were created or analyzed in this study. Data sharing is not applicable to this article.

## Abbreviations

The following abbreviations are used in this manuscript:

APR	Association of Plastic Recyclers
EPA	US Environmental Protection Agency
EPR	Extended producer responsibility
FDA	US Food and Drug Administration
FSDU	Foods for special dietary use
GAO	US Government Accountability Office
GMPs	Good manufacturing practices
HDPE	High-density polyethylene
ISO	International Organization for Standardization
LDPE	Low-density polyethylene
MRF	Materials recovery facility
NIAS	Non-intentionally added substances
PET	Polyethylene terephthalate
PCR	Post-consumer recycled
PP	Polypropylene
US	United States
